# Assessment of heavy metals and their human health risks in selected spices from South Africa

**DOI:** 10.1016/j.toxrep.2023.09.008

**Published:** 2023-09-11

**Authors:** O.M. Oladeji, O.A. Aasa, O.A. Adelusi, L.L. Mugivhisa

**Affiliations:** a Department of Biology and Environmental Sciences, School of Science and Technology, Sefako Makgatho Health Sciences University, Ga-Rankuwa 0208, South Africa; b Department of Biotechnology and Food Technology, Faculty of Science, University of Johannesburg, P.O. Box 17011, Doornfontein Campus, 208 Johannesburg, South Africa

**Keywords:** Heavy metals, ICP-MS, Health risks, Microwave digestion, Spices

## Abstract

The concerns of food safety are rising in developing countries such as South Africa as a result of heavy metal contamination of culinary herbs and spices. Spices and herbs are used for therapeutic purposes as well as flavoring and coloring food. Heavy metals in spices represent significant health risks due to their high toxicity in high quantities. A total of 20 spices samples were purchased from different registered shops, for heavy metals analysis. The samples were prepared, digested, and analyzed with an inductively coupled plasma mass spectrometer (ICP-MS). To ensure the method's accuracy, Polish Certified Reference Mixed Polish Herbs (INCT-MPH-2) from the Food and Drugs Control Center, Poland, was analyzed. The concentrations of Fe (32.22 ± 1.22–131.1 ± 3.26 mg/kg), As (ND to 0.12 ± 0.04 mg/kg), Cr (0.08 ± 0.01–3.2 ± 0.09 mg/kg), Pb (N.D - 0.21 ± 0.02 mg/kg) and Cd (ND to 0.14 ± 0.08 mg/kg) mg/kg were measured. The results revealed that the concentrations of Cr in all spice samples tested were much higher than the Maximum permissible limit (MPL) values. All spices in this study had THQ and HI values less than one, indicating that consumers will experience no potential health hazards from consuming specific metals through spices. However, continual scrutiny should be maintained.

## Highlights

• Spices are the dried parts of plants used historically to enhance food's flavor, color, and aroma. .
• The species samples were digested using a microwave acid-assisted system.
• 5 heavy metals were determined simultaneously by ICP-MS.
• ICP-MS demonstrated high sensitivity and confirmatory capacity needed for determining heavy metals.
• The validated method was applied successfully to spice samples from South Africa.

## Graphical Abstract

**Figure:**
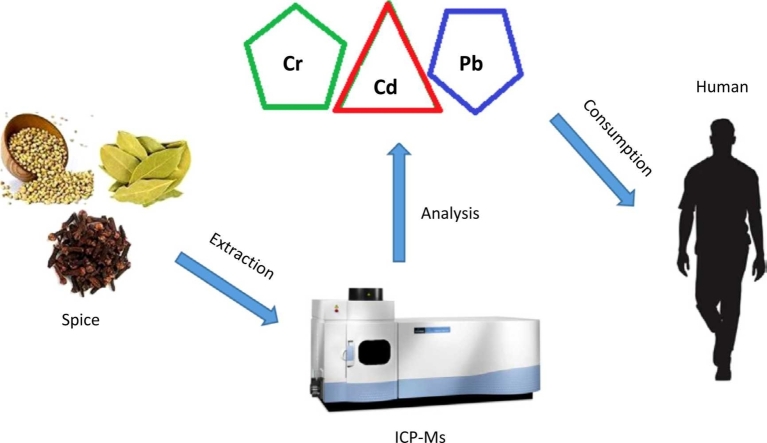

## 1 Introduction

Spices are organic food additives that have been used to improve the sensory quality of foods for thousands of years. Spice roots, barks, fruits, and seeds are mostly used as ingredients in cooking processes to improve the smell, flavor, and color of food, as well as to hide unpleasant odors [19], [30]. In terms of exports, South Africa ranked 10th in the world in 2018, accounting for 2.3% of total exports (saffron, ginger, curry, turmeric, bay leaves, thyme, and other spices) [4]. Information about food composition is crucial for nutritional planning and provides information for epidemiological studies. The primary cause of heavy metal contamination in the food chain is environmental pollution [16]. Individual foods' heavy metal content varies and depends on the heavy metals added during food production, transportation, processing, and fortification [25]. The total amount of heavy metals in the food can be significantly increased by the additional technological procedures used to deliver it to consumers.

Spices and medicinal plants contain heavy metals that are essential to living cells. Understanding the content of trace metals in foods is critical because of their essential or hazardous effects on human health [7]. Heavy metals are non-essential for our bodies even in small amounts, and they are also dangerous because they bioaccumulate. Bioaccumulation, as opposed to chemical concentration in the environment, is the gradual increase in a chemical's concentration in a biological organism [34]. The regular consumption of spices tainted with heavy metals can cause metals to accumulate in the organs of the human body [1], as a result of the potential short-, medium-, and long-term health risks associated with heavy metal accumulation, it is advised to closely monitor these contaminants on a regular basis [24]. Heavy metals may enter the food chain through mineralization by crops or environmental contamination, such as the use of agricultural inputs (i.e pesticides and fertilizers or irrigation from polluted rivers) [1]. Overly high concentrations of heavy metals have a negative impact on human health and can cause birth defects, premature labor, abortion, and mental retardation in children. Adults may also develop neurological issues, tiredness, and excessive blood pressure. Because of the large amount of spices ingested, knowing the harmful metal concentrations of these spices is critical.

Spices are the dried parts of plants that have been used historically to enhance food's flavor, color, aroma, and acceptability [12]. The majority of these have strong scents and flavors. Spices may also be used for medical purposes, religious rituals, cosmetics, or the creation of perfumes [23]. They are made up of the plant's rhizomes, barks, leaves, fruits, seeds, and other components. Due to their widespread approval as a flavoring and condiment in human diets as well as a means of treating illnesses, spices have played an important part in civilization, exploration, and commerce throughout history [27], [8]. However, the handling and preparation steps can turn them into a source of food poisoning. Additionally, the use of spices has significantly increased in most parts of the world over the past three decades, largely due to their medicinal properties. Several herbs and spices are either grown naturally in various regions or are grown on small farms.

Regarding heavy metal contamination, there is limited information available about the safety of spices, according to the literature, Pb was found in unbranded turmeric samples and was found to be above the permissible limit [31]. Other studies examined the concentration of heavy metals in spice samples and showed that the majority of heavy metal concentrations in the spices examined were under permitted levels and safe for human consumption [11], due to the importance of this research and its significant connection to human health, the study aims to determine the level of heavy metals in 20 commonly used spices sold at registered stores in South Africa, as well as to estimate health hazard concerns associated with heavy metal intake through spice consumption.

## 2 Materials and methods

### 2.1 Sample collection and processing

A total of twenty common spices were randomly bought from registered shops in South Africa. The samples were kept in zip lock bags and were stored at room temperature, until analysis. The species samples were digested using a microwave acid-assisted system. 0.5 g of spice samples were weighed into Teflon vessels, and 10 mL of 65 % HNO_3_ was then added. According to the manufacturer's recommendations, the vessels were heated in a microwave system to a maximum of 1200 W before undergoing a microwave digestion program. Following thorough digestion, the clear solutions were poured into 50-mL volumetric flasks, filtered through Whatman No. 1 filter paper (Whatman Ltd., England), and then made up to 50 mL using ultra-pure de-ionized water. The concentrations of Fe, Cd, As, Cr, and Pb were determined using ICP-MS NexION 300Q from PerkinElmer, USA. The analysis was carried out in accordance with the following procedure: sample introduction, fragmentation of compounds with a characteristic ion charge, nebulization, ionization of elements in high temperature argon plasma, detection based on *m/z* ratio, mass discrimination in the quadrupole analyzer, and data analysis as described in the Table 1 below.

**Table 1:** Shows the optimal operating conditions for ICP-MS analysis.

Operational parameters	
Acquisition mode	Jump peak
ICP RF power	1050 W
Plasma gas flow	16 L/min
Scanning time	20 s
Integral time	36 s
Auxiliary gas flow rate	1.2 L/min
Detector	Double mode
Repetition	3 times
Atomizer flow	0.89 L/min
Readings	1
Atomizer	Concentric Nebulizer

Prior to the measurement, the ICP-MS spectrometer was calibrated using a multi-elemental mixture of metal standards (Agilent Technologies, Japan). Each solution was measured three times, and the heavy metals were quantified using external calibration curves. The standard solution of equal concentrations of mixed heavy metals was diluted into seven different concentrations of 1 % nitric acid solution, and each standard solution received 50 μg/L of the internal standard (Sc). The analytical processes' quality was tested using Polish Certified Reference Material Mixed Herbs (INCT-TL-2) that were processed in the same way as the spice samples. A calibration solution was prepared using the ICP-MS multi-element stock standard solution (Merck) and internal standard (Sc) to acquire the sensitivity factors for the individual elements. The calibration curves for all elements show good linearity across the entire concentration range, with determination coefficients greater than 0.999. The limit of quantitation (LOQ) and limits of detection (LOD), as well as recovery was performed.

### 2.2 Health risk assessment of spices consumption

The potential health risks of consuming heavy metals through spices were assessed using the estimated daily intake (EDI), target hazard quotient (THQ), and Hazard Index (HI). The EDI value is determined by the amount consumed daily, body weight, and element concentrations in spices (Eq. (1)).

(1) $$\mathrm{EDI}=\frac{\mathrm{Cmetal}*\mathrm{IR}\;}{\mathrm{BW}*1000}$$

Where,

EDI is estimated daily intake.

C_metal_ (mg/kg) concentration of heavy metal content in spices,

IR (ingestion rate gram/day person) (10 g/day/person),

BW represent the body weight (kg) (60 kg).

THQ is used to assess the non-carcinogenic risks of long-term exposure to contaminants in Spices, and the calculations were done using Eq. (2).

(2) $$\mathrm{THQ}=\frac{\mathrm{EDI}}{\mathrm{RfD}\;}$$

Where, RfD is reference dose values for each metals of interest are; Cd (0.001), As (0.003), Pb (0.004) and Cr (0.003) mg/kg per day, respectively [10].

To calculate the overall non-carcinogenic risk to human health posed by exposure to multiple pollutants, the HI was developed. HI is the sum of the HQ for all heavy metals found in spices, as shown in Eq. (3).

(3) HI=∑THQ

If the values of THQ/HI ≥ 1 imply that the population may have negative health effects, while if THQ/HI < 1, the population is not expected to encounter any obvious negative consequences [39].

## 3 Result and discussion

The heavy metal content of various types of spices available in South African shops was measured in this study. Table 1 shows the optimal operating conditions for ICP-MS analysis used in this study. Observed LOD and LOQ ranged from 0.04 to 1.01 and 0.05 to 1.12, respectively. Table 2 shows the method validation and permissible limits for heavy metals in plants.

**Table 2:** Method validation.

Element	Determined value (mg/kg)	Certified value (mg/kg)	LOD	LOQ	Recovery %	Permissible limit for species (mg/kg)
Pb	1.54 ± 0.24	1.78 ± 0.24	0.04	0.05	87	5
Cr	1.91 ± 0.01	1.91 ± 0.22	0.05	0.13	100	0.3
As	0.11 ± 0.21	0.10 ± 0.02	0.32	0.45	96	0.2
Cd	0.29 ± 0.01	0.03 ± 0.00	0.09	0.19	97	0.2
Fe	423 ± 10.27	432 ± 0.00	1.01	1.12	97	300

The analytical data revealed that all of the samples contained a low concentration of Pb. Pb concentrations ranged from 0.08 ± 0.001 to 2.51 ± 0.01 mg/kg (Table 3), these values were minimal in compared to the FAO/WHO global permitted limit of 5 ppm, and so these spices are not considered a concern to human health. In comparison to current studies with other nations, we found that Pb concentrations in Romania, Pakistan, and Iraq varied from 0.04 to 1.28 mg/kg [21], 4.44 to 15.88 mg/kg [2], and 3.21 to 6.98 mg/kg [15], respectively. Pb is a toxic heavy metal that binds to oxo-groups in enzymes and interferes with almost every step of hemoglobin synthesis and porphyrin metabolism [24]. Pb toxicity in humans has been linked to mental retardation, encephalopathy seizures, and other health problems. Due to its high toxicity, Pb is a cumulatively toxic substance that harms a variety of human body systems, especially young children [5], it poses a threat to the health and safety of living things and because they can accumulate and concentrate it, very small concentrations of it are thought to be extremely dangerous [29]. In relation to protein uptake sites in the mucous membrane of the small intestine, Pb metal is seen as a direct competitor to Ca. Pb poisoning causes deficiencies in the thyroid gland's function and causes mental retardation in children because it is concentrated in the brain, bones, blood, kidneys, thyroid gland and blood [38].

**Table 3:** Concentrations (mg/kg) of heavy metals from spices samples marketed in South Africa.

Sample I.D	Pb	Cr	As	Cd	Fe
1	0.11 ± 0.02	1.91 ± 0.14	N.D	0.14 ± 0.08	210 ± 4.51
2	0.07 ± 0.01	3.21 ± 0.09	N.D	< LOD	32 ± 1.22
3	0.11 ± 0.00	2.53 ± 0.05	< LOD	< LOD	90 ± 1.60
4	0.13 ± 0.00	2.11 ± 0.04	N.D	< LOD	75 ± 1.70
5	N.D	1.80 ± 0.06	0.09 ± 0.10	N.D	117 ± 2.60
6	0.06 ± 0.02	0.73 ± 0.04	< LOD	N.D	82 ± 2.78
7	0.19 ± 0.00	0.26 ± 0.04	< LOD	N.D	110 ± 3.41
8	N.D	0.46 ± 0.06	N.D	N.D	162 ± 3.33
9	0.16 ± 0.00	1.94 ± 0.05	N.D	N.D	93 ± 1.92
10	0.08 ± 0.01	2.98 ± 0.15	0.12 ± 0.04	N.D	112 ± 1.34
11	0.11 ± 0.01	2.07 ± 0.14	N.D	0.11 ± 0.01	103 ± 1.34
12	0.15 ± 0.00	1.42 ± 0.07	N.D	N.D	192 ± 4.85
13	0.12 ± 0.02	1.80 ± 0.06	N.D	N.D	131 ± 3.26
14	0.20 ± 0.02	0.31 ± 0.02	N.D	< LOD	121 ± 3.19
15	0.17 ± 0.02	0.12 ± 0.01	N.D	< LOD	54 ± 0.80
16	0.11 ± 0.01	0.08 ± 0.01	N.D	< LOD	106 ± 2.68
17	N.D	0.18 ± 0.01	N.D	N.D	127 ± 5.33
18	0.21 ± 0.02	1.32 ± 0.10	N.D	N.D	162 ± 3.10
19	0.04 ± 0.02	1.65 ± 0.04	N.D	N.D	93 ± 2.94
20	0.13 ± 0.01	0.89 ± 0.06	N.D	< LOD	104 ± 2.71

Cr highest mean concentration were found to be 3.2 ± 0.09 mg/kg and lowest mean concentrations were 0.08 ± 0.01 mg/kg as presented in Table 3. When compared the present studies with other studies, we find that concentrations of Cr from Nigeria varied from 0.34 mg/kg to 0.89 mg/kg [9]. Cr is a toxic heavy metal that, when kept below bearable levels, plays a crucial role in the physiological and biological functioning of the human body [17]. Cr is found in all plants but is not required; there is no absorption pathway for its uptake, so it is dependent on Cr speciation [37]. Plants do not take this metal directly, but rather acquire it via carrier ions such as sulfates or ferrous ions. Its toxicity interferes with photosynthesis, other metabolic processes, and total dry matter production, causing plant germination and growth to be disrupted [35]. However, a lack of it causes hyperglycemia and an increase in body fat, while a high Cr content damages the liver, liver, and blood cells through oxidation processes [13].

The presence of As was found only in 2 samples (2 %) with the concentration ranges from N.D - 0.12 ± 0.04 mg/kg. The amount of the metal that was being measured in each of the samples that were analyzed fell below the permissible limit (0.2 mg/kg). Similar report of As was discovered ranging from 0.03 mg/kg and 0.06 mg/kg in nutmeg and black pepper samples to 0.37 mg/kg in oregano samples [33]. The level of As contamination in the present research results was similar to that obtained in Poland [20] and in other EU countries (0.066 mg/kg) [3], but significantly lower than the findings of studies that have been carried out in Asia, where the levels of As in various types of tea ranged from below the detection threshold to 4.81 mg/kg [14], [36], [41].

The concentration of Cd ranged between N.D to 0.14 ± 0.08 mg/kg while permissible limit of Cd is 0.2 mg/kg in plant [28]. The presented study were compared with a study from Poland and there result revealed that Cd was low and varied from < LOQ (0.020 mg/kg) to 0.082 mg/kg [22]. None of their analyzed samples contained Cd levels exceeding the permitted limits for that metal. The results obtained in this study and the results published in the literature are similar levels of Cd in samples of herbs and spices analyzed.

Fe is a significant trace element, and mixtures of iron and proteins are essential for metabolism in all living things. According to analytical findings, the Fe content of spice samples ranged from 32 ± 1.22 to 131.1 ± 3.26 mg/kg (Table 3). Despite the low levels of Fe in all the samples. All of the outcomes fell within the 300 mg/kg FAO/WHO permissible limits. When comparing the findings of this study with those of a study done on spices in Tikrit and Baghdad city, we found that the iron concentration ranged between (6.870–19.370 mg/kg) [11] and (32–490 mg/kg) [32], it can be seen that their study were similar to the present study.

Fe is a component of the active site of several reproductive hydrogenases and is most frequently linked to ligands that contain sulfur [26]. Fe is a necessary element. Fe plays a crucial part in metabolism along with ferrodoxin, hemoglobin, and other components. Fe promotes the oxidation of fat, protein, and carbohydrates to regulate body weight, which is a key factor in some diseases (diabetes) [18]. Anemia is one of the health problems brought on by a low iron level in the blood, but an excess of iron can also cause cirrhosis, heart failure, and diabetes. Early signs of iron poisoning include stomach pain, vomiting and nausea [6]. The wide distribution of iron in the soil and the regional variations in air pollution with this metal are likely to be the causes of the variation in Fe concentration. It is also possible that the pesticides used or the iron metal that was absorbed during the grinding of these spices are to blame.

### 3.1 Health risk assessment

The EDI values of toxic metals through the consumption of spices are summarized in Table 4. The EDI values of Pb, Cr, As, Cd and Fe were calculated to be 0.000135–0.000093, 0.00003–0.0000517, 0.000012–0.000015, 0.0000185–0.0000233, and 0.015–0.000053, respectively. The trends of EDI values were in the order of Fe > Cr > Pb > Cd > As, indicating that Pb would increase the amount of heavy metals consumed each day. EDI values typically had the same levels for As and Cd. The consumption of the spices under study does not seem to pose a health risk, as shown by the fact that all of the EDI values in this study fall below the RfD values for all elements according to Table 4.

**Table 4:** Estimated daily intake of the heavy metals analyzed.

Sample I.D	Pb	Cr	As	Cd	Fe
1	1.35 × 10^−4^	3.18 × 10^−4^	-	2.33 × 10^−5^	3.5 × 10^−2^
2	7.83 × 10^−5^	5.35 × 10^−4^	-	-	5.33 × 10^−3^
3	1.83 × 10^−5^	4.22 × 10^−4^	-	-	1.5 × 10^−2^
4	1.88 × 10^−4^	3.52 × 10^−4^	-	-	1.25 × 10^−2^
5	4.18 × 10^−4^	3 × 10^−4^	1.50 × 10^−5^	-	1.95 × 10^−2^
6	9.33 × 10^−5^	1.22 × 10^−4^	-	-	1.37 × 10^−2^
7	8.17 × 10^−5^	4.33 × 10^−5^	-	-	1.83 × 10^−2^
8	2.17 × 10^−5^	7.66 × 10^−5^	-	-	2.7 × 10^−2^
9	4.33 × 10^−5^	3.23 × 10^−4^	-	-	1.55 × 10^−2^
10	1.33 × 10^−5^	4.97 × 10^−4^	1.20 × 10^−5^	-	1.86 × 10^−2^
11	8.5 × 10^−5^	3.45 × 10^−4^	-	1.85 × 10^−5^	1.72 × 10^−2^
12	4.17 × 10^−5^	2.37 × 10^−4^	-	-	3.2 × 10^−2^
13	2 × 10^−5^	3 × 10^−4^	-	-	2.18 × 10^−2^
14	3.33 × 10^−5^	5.17 × 10^−5^	-	-	2.02 × 10^−2^
15	6.17 × 10^−5^	2 × 10^−5^	-	-	9 × 10^−3^
16	1.02 × 10^−4^	1.33 × 10^−5^	-	-	1.77 × 10^−2^
17	6.66 × 10^−5^	3 × 10^−5^	-	-	2.12 × 10^−2^
18	2.02 × 10^−4^	2.2 × 10^−4^	-	-	2.7 × 10^−2^
19	1.4 × 10^−4^	2.75 × 10^−4^	-	-	1.55 × 10^−2^
20	1.22 × 10^−4^	1.48 × 10^−4^	-	-	1.73 × 10^−2^

All spices had THQ values that were less than one, indicating that there is no potential health risk associated with consuming spices. Table 5 demonstrates that the study's individual toxic metal THQ values for ingestion of spices were less than 1. This finding implies that it is unlikely that potentially dangerous daily ingestion of specific toxic metals through the use of spices will take place. The HI value can be used to express the combined non-carcinogenic effects of Pb, Fe, Cr, Cd, and As. For every metal examined through the consumption of spices (Table 5), the HI values were under 1. This shows that there are no significant non-carcinogenic health risks for the consumers [40].

**Table 5:** Target hazard quotient and hazard index.

Sample I.D	Pb	Cr	As	Cd	Fe	HI
1	3.37 × 10^−2^	1.06 × 10^−1^	-	2.33 × 10^−2^	5 × 10^−2^	0.19
2	1.96 × 10^−2^	1.78 × 10^−1^	-	-	7.62 × 10^−3^	0.23
3	4.58 × 10^−3^	1.41 × 10^−1^	-	-	2.14 × 10^−2^	0.17
4	4.71 × 10^−2^	1.17 × 10^−1^	-	-	1.79 × 10^−2^	0.18
5	1.04 × 10^−1^	1 × 10^−1^	5 × 10^−3^		2.78 × 10^−2^	0.24
6	2.33 × 10^−2^	4.05 × 10^−2^	-	-	1.9 × 10^−2^	0.08
7	2.04 × 10^−2^	1.44 × 10^−2^	-	-	2.61 × 10^−2^	0.06
8	5.42 × 10^−3^	2.55 × 10^−2^	-	-	3.86 × 10^−2^	0.07
9	1.08 × 10^−2^	1.08 × 10^−2^	-	-	2.21 × 10^−2^	0.14
10	3.33 × 10^−2^	1.66 × 10^−1^	6.66 × 10^−2^	-	2.67 × 10^−2^	0.26
11	2.12 × 10^−2^	1.15 × 10^−1^	-	1.83 × 10^−3^	2.45 × 10^−2^	0.16
12	1.04 × 10^−2^	7.89 × 10^−2^	-	-	4.57 × 10^−2^	0.15
13	5 × 10^−3^	1 × 10^−1^	-	-	3.12 × 10^−2^	0.14
14	8.33 × 10^−3^	1.72 × 10^−2^	-	-	2.88 × 10^−2^	0.05
15	1.54 × 10^−2^	6.67 × 10^−3^	-	-	1.28 × 10^−2^	0.03
16	2.54 × 10^−2^	4.44 × 10^−3^	-	-	2.52 × 10^−2^	0.05
17	1.67 × 10^−2^	1 × 10^−2^	-	-	3.02 × 10^−2^	0.06
18	5.04 × 10^−2^	7.33 × 10^−2^	-	-	3.86 × 10^−2^	0.16
19	3.5 × 10^−2^	9.17 × 10^−2^	-	-	2.21 × 10^−2^	0.15
20	3.04 × 10^−2^	4.94 × 10^−2^	-	-	2.48 × 10^−2^	0.10

## 4 Conclusion

In conclusion, the purpose of this study was to determine the amount of heavy metals present in some selected South African spices. Applying the optimized acid digestion method, which was validated through a recovery experiment, yielded good percentage recoveries (87–100 %) for the analysis of heavy metals in spices. All of the heavy metals investigated through the consumption of spice samples had HI values lower than 1. This shows that using these spices in different homes poses no significant non-carcinogenic health risk. Additionally, it is advised to discard the first infusion and draw from the second to reduce the amount of toxic metals that consumers consume in order to counteract the effects of the concentrations of toxic metals. Finally, it is proposed that heavy metals be continuously monitored in all food commodities in order to assess health risks from toxic heavy metals and safeguard consumers.

## CRediT authorship contribution statement

**O.M. Oladeji:** Visualization, Project administration, Supervision, Validation, Data curation, Writing - original draft, Writing - review & editing. **O.A. Aasa:** Visualization, Software, Writing - review & editing. **O.O. Adelusi:** Visualization, Software, Methodology, Writing - review & editing. **L.L. Mugivshisa:** Project administration, Supervision, Validation, Funding acquisition, Writing - review & editing.

## Declaration of Competing Interest

The authors declare that they have no known competing financial interests or personal relationships that could have appeared to influence the work reported in this paper.

## Data Availability

Data will be made available on request.
